# Porous Biocoatings Based on Diatomite with Incorporated ZrO_2_ Particles for Biodegradable Magnesium Implants

**DOI:** 10.3390/jfb14050241

**Published:** 2023-04-24

**Authors:** Mariya B. Sedelnikova, Alexander D. Kashin, Pavel V. Uvarkin, Alexey I. Tolmachev, Yurii P. Sharkeev, Anna V. Ugodchikova, Nikita A. Luginin, Olga V. Bakina

**Affiliations:** 1 Laboratory of Physics of Nanostructured Biocomposites, Institute of Strength Physics and Materials Science of SB RAS, Tomsk 634055, Russia; kash@ispms.ru (A.D.K.); uvarkin@ispms.tsc.ru (P.V.U.); tolmach@ispms.ru (A.I.T.); sharkeev@ispms.ru (Y.P.S.); ugodch99@gmail.com (A.V.U.); nikishek90@gmail.com (N.A.L.); 2 Research School of High-Energy Physics, National Research Tomsk Polytechnic University, Tomsk 634050, Russia; 3 Laboratory of Plasma Synthesis of Materials, Troitsk Institute for Innovation & Fusion Research, Troitsk 108840, Russia; 4 Laboratory of Nanobioengineering, Institute of Strength Physics and Materials Science of SB RAS, Tomsk 634055, Russia; ovbakina@ispms.tsc.ru

**Keywords:** magnesium implants, micro-arc coating, diatomite, ZrO_2_ particles, corrosion resistance

## Abstract

In the present work, the surface of a biodegradable Mg alloy was modified to create porous diatomite biocoatings using the method of micro-arc oxidation. The coatings were applied at process voltages in the range of 350–500 V. We have studied the influence of the addition of ZrO_2_ microparticles on the structure and properties of diatomite-based protective coatings for Mg implants. The structure and properties of the resulting coatings were examined using a number of research methods. It was found that the coatings have a porous structure and contain ZrO_2_ particles. The coatings were mostly characterized by pores less than 1 μm in size. However, as the voltage of the MAO process increases, the number of larger pores (5–10 μm in size) also increases. However, the porosity of the coatings varied insignificantly and amounted to 5 ± 1%. It has been revealed that the incorporation of ZrO_2_ particles substantially affects the properties of diatomite-based coatings. The adhesive strength of the coatings has increased by approximately 30%, and the corrosion resistance has increased by two orders of magnitude compared to the coatings without zirconia particles.

## 1. Introduction

Over the past few years, significant progress has been made in the fields of bioengineering and orthopedics. Presently, there are a number of ways to treat bone-related diseases and defects. These include autologous bone grafting, the induced membrane technique, distraction osteogenesis, etc. [1,2,3,4,5,6,7,8]. However, the method of installing various types of implants—screws, plates, etc.—remains the most popular and convenient. Depending on their interaction with human body tissues, metal orthopedic implants can be generally divided into two main groups: bioinert and biodegradable [9,10,11,12,13]. Bioinert implants do not react with surrounding tissues in the human body. Bioinert materials for manufacturing implants include titanium (Ti) and its alloys, as well as some steels [14,15,16,17,18,19]. Their benefits include high specific strength and good biocompatibility. The main disadvantages of such materials are their high elastic modulus, which can lead to stress shielding of the bone, and the need for secondary surgery to remove the overdue implant [20,21]. Thus, nowadays more and more attention is being given to bioresorbable materials that have the ability to dissolve in the human body without releasing any kind of toxic elements.

There is a wide variety of biodegradable materials used for bone repair. They can be divided into three main groups: ceramics, polymers, and metals [22,23,24,25]. However, as with any material, it has its downsides. For instance, Song et al. have reported that biodegradable polymers are highly physiologically active and have low mechanical properties [26]. Ceramic implants have low fracture toughness, extremely high stiffness, and are brittle, as demonstrated by Alizadeh-Osgouei et al. [27]. Bioresorbable metals, namely Mg, have a very high rate of biodegradation and are more susceptible to corrosion than their bioinert counterparts, as can be concluded from Chakraborty Banerjee’s et al. review [28]. Another drawback of an Mg implant is the intensive release of hydrogen at the implantation site, which disrupts the natural pH balance of the human physiological medium and inflames the surrounding tissues [29,30]. In some cases, this can potentially lead to rejection of the implant. However, magnesium-based alloys are still considered to be one of the most promising materials for treating bone defects and regenerating bone tissue. The advantages of Mg-based alloys include superior mechanical properties compared to biodegradable polymers and ceramics. The density of magnesium-based alloys is closely matched with the density of human bone. Moreover, Young’s modulus of Mg is also relatively similar to that of human bone, which reduces the risk of stress shielding, as stated in the Chen et al. review article [31].

To eliminate the disadvantages of magnesium, we have modified the surface of an Mg alloy by synthesizing a protective, biologically active coating based on diatomite with the inclusion of ZrO_2_ particles. Such a coating not only protects the implant from dissolving too quickly in a human body but also contributes to the activation of the processes of osteosynthesis and facilitates bone tissue regeneration, as described in our previous research paper [32].

The method of micro-arc oxidation (MAO, or plasma electrolytic oxidation—PEO) is one of the most cost-efficient and versatile ways of generating a protective coating on the surface of valve metals [33]. The MAO process is represented by the occurrence of a large number of micro-arc discharges developing on the metal surface under the influence of a strong electric field. In the zone of dielectric breakdown, the materials of the substrate, oxide layer, and electrolyte shift into a plasma state and interact with each other, leading to the formation of a dielectric coating, as outlined by Simchen et al. and Clyne et al. [34,35].

Nowadays, a wide variety of micro-arc coatings are being developed by various researchers. Changing the compositions of electrolytes for synthesizing the coatings leads to the formation of new coatings with different structures, properties, and functions. The most popular micro-arc coatings today are based on calcium phosphates (brushite, hydroxyapatite, tricalcium phosphate), calcium silicates (wollastonite, akermanite), etc. [36,37,38,39,40,41,42,43,44].

Various properties of these coatings can be tailored and improved by adding nano- and micro-particles of different refractory oxides, such as ZrO_2_, SiO_2_, TiO_2_, Al_2_O_3_ and several others [45,46,47,48,49,50,51,52]. In this study, we focus on incorporating zirconia into the structure of diatomite-based micro-arc coatings, which were studied in detail in our previous work [32], by adding the microparticles to the electrolyte solution. The resulting coatings have been examined by a number of methods to assess their morphology as well as various physical, mechanical, chemical, and biological properties.

The main purpose of this work is to study the influence of ZrO_2_ particles added to diatomite-based bioactive coatings for magnesium implants on their morphology, phase composition, physical properties, mechanical strength, biocompatibility, and corrosion resistance.

## 2. Materials and Methods

A high-purity Mg alloy MA2-1hp (JSC “VILS”, Moscow, Russia) was used as a substrate material. This alloy is equivalent to the widely used AZ31 magnesium alloy, and its chemical composition is as follows: ≈94% Mg, ≈3.8% Al, ≈1.0% Zn, ≈1.2% other elements combined; 10 × 10 × 1 mm samples were cut out by the method of electric discharge machining (EDM). The samples were sanded with 600-grit corundum sandpaper and subsequently cleaned in an ultrasonic bath (Elmasonic S, Elma, Singen, Germany) prior to the MAO treatment. The electrolyte consisted of the following soluble components: sodium hydroxide (NaOH), sodium silicate (Na_2_SiO_3_), and sodium fluoride (NaF). In addition, particles of two types of refractory compounds, zirconia (ZrO_2_) and diatomite (SiO_2_ · nH_2_O), were added to this solution. ZrO_2_ had a monoclinic modification and an average particle size of 1–2 µm. Diatomite is a sedimentary rock of biogenic origin consisting mainly of the shells of diatom algae, which, in turn, consist of amorphous silica. A more detailed description was given in our previous paper [32].

The prepared Mg samples were coated by the method of micro-arc oxidation (MAO). The parameters of the coating process were as follows:

- Anodic potentiostatic mode;
- 50 Hz pulse frequency;
- 100 µs pulse duration;
- 350–500 V voltage range (50 V step);
- 5 min deposition duration.

Further details of the setup and the process were described previously in [32].

The surface morphology of the resulting coatings was studied using the LEO EVO 50 electron microscope (Zeiss AG, Oberkochen, Germany). The elemental composition of the coatings was examined using the energy dispersive X-ray spectroscope (Oxford Instruments, Abingdon, UK).

The phase compositions of both the initial zirconia powder and the resulting coatings were determined using the DRON–7 X-ray diffractometer (Burevestnik, Nizhniy Novgorod, Russia). The X-ray examination was performed with the following imaging parameters: Co K_α_ radiation, 35 kV voltage, 22 mA tube current, and a range of 2*θ* angles from 10° to 90° with a scanning step of 0.02°.

The porosity of the samples was calculated by the following Equation (1):

(1) $$P\;\left(\%\right)=\frac{\sum l}{\sum L}\times 100,$$

where *L* is the full length of secants on the SEM images and *l* is the length of the secants within the pores, as described in previous work [21]. The sizes of structural elements were measured by the secant method using SEM images according to ASTM E1382-9 and DD ENV 1071-5.

The scratch testing of the samples was performed using the Macro Scratch Tester Revetest^®^ RST (CSM Instruments, Peseux, Switzerland), equipped with a diamond indenter of 200 µm in radius. The indenter was moved over the surface of the sample at a speed of 1 mm/min with a linearly increasing load, from 0.5 to 30 N. The load speed was 5.9 N/min. The length of the track was 5 mm.

The electrochemical activity of the resulting samples was assessed using the P-40X pulse potentiostat-galvanostat (Electrochemical Instruments, Chernogolovka, Russia). A three-electrode cell with a 0.9% NaCl solution was used for conducting the experiment. An Ag/AgCl electrode acted as a reference electrode, while a graphite rod was used as a counter electrode. The exposed sample surface area was 1 cm^2^. The potentiodynamic polarization (PDP) curves were obtained at 2 mV/s in a ±1.0 V range of electrode potential.

The study of the elastoplastic properties and microhardness of the coatings was carried out by the method of indentation via the Duramin 5 microhardness tester (Struers, Copenhagen, Denmark) at a load of 500 mN for 10 s.

The process of bioresorption of the samples was carried out in a 0.9 wt.% sodium chloride solution (ISO 10993-15-2011). The samples were held in the solution for 11 days at a temperature of 37 °C to simulate the internal temperature of the human body. The mass loss of the samples Δ*m* was calculated by the following Equation (2):

(2) $$\Delta m=\frac{m_{o}-m_{i}}{m_{0}}\cdot 100,\;\%$$

where *m*_0_ is the mass before dissolution, mg, *m_i_* is the mass after dissolution, mg. The sample surfaces before and after dissolution were inspected via the MET 1MT optical microscope (Altami, Saint Petersburg, Russia).

The cytotoxic properties of the coatings were studied via the MTT assay. In this case, the NIH/3T3 mouse cell culture obtained from the SRC VB “VECTOR” (Novosibirsk, Russia) was used. The cells were cultivated as monolayers in DMEM medium supplemented with 10% fetal bovine serum, 2 mM l-glutamine, and 1% penicillin/streptomycin (HyClone, Logan, UT, USA). Cells were cultured for 24 h in a humidified environment of 95% air and 5% CO_2_ at 37 °C. The final cell concentration was 1 × 10^4^ cells per 100 µL of medium in a 96-well microplate well (TPP, Trasadingen, Switzerland). The test specimens were extracted for 24 h at 37 °C at a surface-to-volume ratio equal to 1 cm^2^/mL of DMEM medium. 

The obtained cell suspensions were used for the MTT test, which is based on the reduction of MTT reagent by live cell reductases to formazan stained purple. The incubation with MTT solution was carried out at 37 °C and 5% CO_2_ for 2 h. The supernatants optical density was estimated using the Thermo Scientific Multiskan FC microplate spectrophotometer (Thermo Fisher Scientific, Waltham, MA, USA) at a wavelength of 570 nm.

## 3. Results

### 3.1. Coating Thickness and Roughness

**Figure 1:**
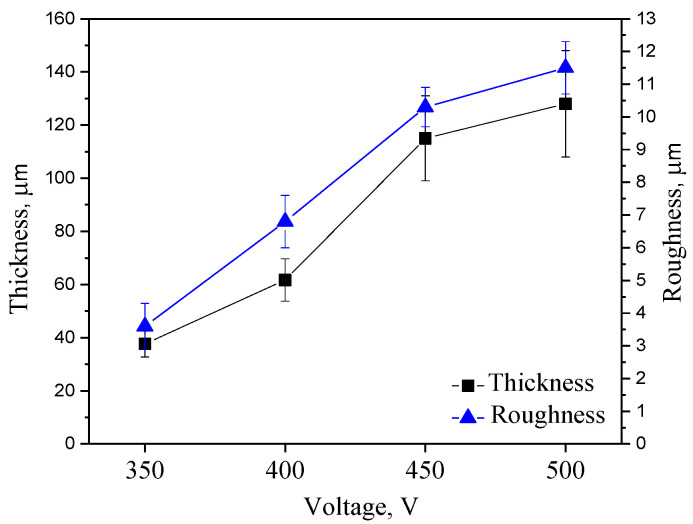
Coating thickness (black line) and roughness (blue line) in relation to the deposition process voltage in the range of 350–500 V.

It can be seen that the thickness of the coatings increases correspondingly with an increase in MAO voltage and ranges from approximately 40 µm at 350 V to 130 µm at 500 V (Figure 1). This correlation is typical for micro-arc coatings and aligns well with previous studies on the topic [53,54]. However, there is a large scatter in the data (the average deviation is high). In addition, an intensive development of surface roughness should be noted. At a process voltage of 350 V, the roughness value of Ra was approximately 3.5 μm, and at 400 V the roughness increased to 6.7 μm (Figure 1). With a further increase in voltage to 450–500 V, the surface roughness increased up to 11.5 µm.

### 3.2. SEM

#### 3.2.1. Coating Surface Morphology

**Figure 2:**
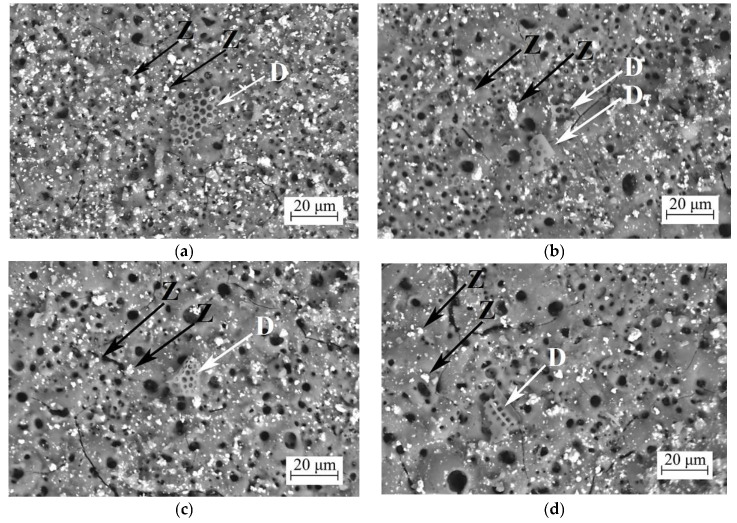
SEM images of the coatings with ZrO_2_ particles deposited at different voltages—(**a**) 350 V, (**b**) 400 V, (**c**) 450 V, and (**d**) 500 V. (Z) Black arrows—ZrO_2_ particles; (D) White arrows—diatomite particles.

The morphology of the coatings can be observed in Figure 2. It can be seen that the coatings have a complex structure: two types of particles on the surface, as well as pores formed both as a result of micro-arc discharges and inherited from the mesh structure of diatoms when they were partially fused into the coating. Remains of diatom algae skeletons are observed on the surface of the coatings (Figure 2, marked “D” with a white arrow). We can also observe the inclusion of a large number of ZrO_2_ particles on the surface layer of the coating (Figure 2, marked “Z” with a black arrow). The coatings are dominated by pores with sizes under 1 μm. However, as the voltage of the MAO process increases, the number of larger pores with sizes of 5–10 μm increases as well (Figure 2c,d). In addition, there is a decrease in the number of particles on the coating surface. It should also be noted that the relief of the coatings becomes more pronounced. The surface porosity of the coatings with ZrO_2_ particles remains consistent regardless of the deposition process voltage and has a value of approximately 4.5–5.0%. The coatings without the ZrO_2_ particles, studied previously [32], were generally more porous, but the porosity decreased as the MAO voltage increased and ranged from 23% at 350 V to 9.5% at 500 V.

#### 3.2.2. Coatings Cross-Sections

**Figure 3:**
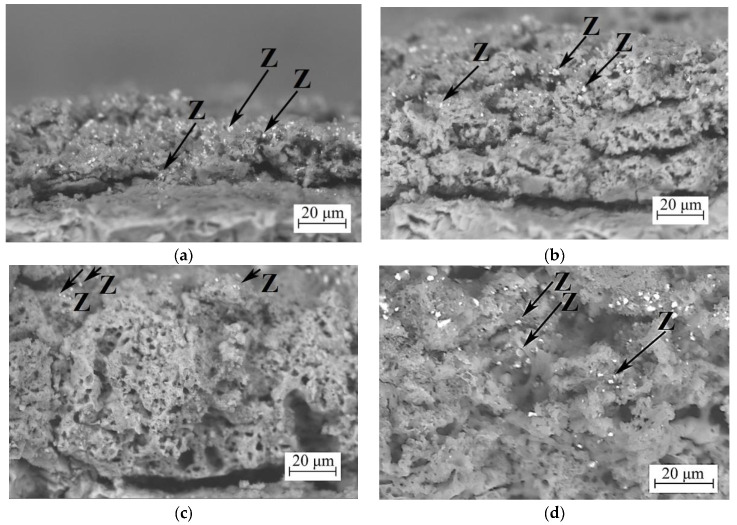
SEM images of the cross section of the coatings with ZrO_2_ particles deposited at different voltages—(**a**) 350 V, (**b**) 400 V, (**c**) 450 V, and (**d**) 500 V. (Z) Black arrows—ZrO_2_ particles.

Figure 3 shows cross-sections of the coatings. The cross-sectional images of the coatings show that the coatings are porous and contain a large number of open, closed, and channel pores. They are the result of the implementation of different types of micro-arc discharges (cascade multiple and single powerful discharges [34,55]). In addition, small pores are inherited from the porous cell structure of diatomite [32]. However, diatomite particles are not observed in the cross-sections of the coatings. As for particles of zirconium dioxide, small amounts are observed in the deep layers of the coatings deposited at 350 V (Figure 3a). At higher MAO process voltages (400–500 V), the particles remain only on the surface layers of the coatings (Figure 3c,d). Zirconium dioxide is known to have a very high melting point of 2680 °C [56]. However, it is possible that at high voltages in the MAO process, even higher temperatures develop in the micro-arc discharge channels and the melting of zirconia particles takes place.

It can also be observed that the pores remain relatively consistent in size all throughout the thickness of the coating, unlike the coatings without the addition of zirconia particles, where the pores were decreasing in size towards the substrate [32].

### 3.3. EDX Results

#### 3.3.1. Coatings Surface

**Figure 4:**
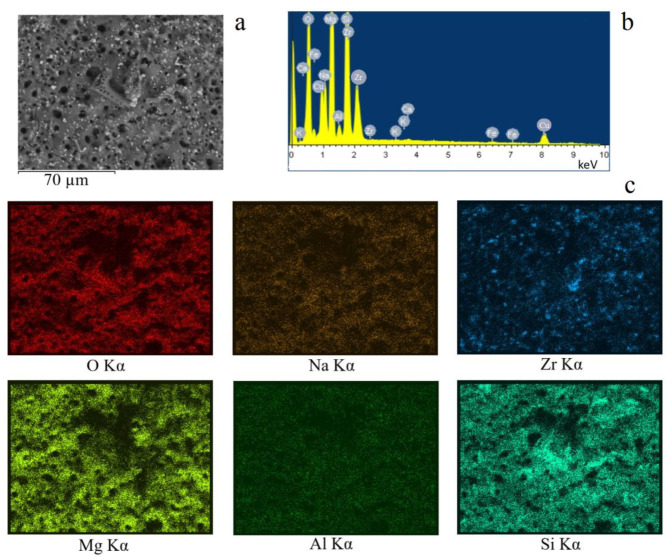
SEM image of the (**a**) coating, (**b**) the EDX spectrum, and (**c**) the maps of element distribution across the surface of the coating.

To reveal the chemical composition and the distribution of the elements across the surface of the coating, an energy dispersive X-ray (EDX) analysis was carried out. It has been revealed that the key elements comprising the coating are O, Mg, Si, Zr, and Na (Figure 4, Table 1). The content of other elements (Al, Ca, and Fe) does not exceed 1.0 at.%. All key elements were distributed evenly across the coating surface, with the exception of zirconia, which had higher localized concentrations in the areas where the non-melted particles were located (Figure 4c). It should also be noted that the coating surface had elevated levels of silica in the areas where the unmelted diatomeae remained.

Table 1 summarizes the quantitative content of each of the elements on the surface of the coatings, depending on the voltage of the deposition process. It can be observed that both magnesium and silicon content increase, whereas zirconium content decreases with increasing MAO voltage. This can be attributed to the more intensive flow of the processes of interaction between the substrate material and the electrolyte and the increase in the thickness of the coatings. On the other hand, an increase in the voltage of the MAO process was accompanied by an increase in the intensity of micro-arc discharges, during which more ZrO_2_ particles were melted.

#### 3.3.2. Coatings Cross-Sections

**Figure 5:**
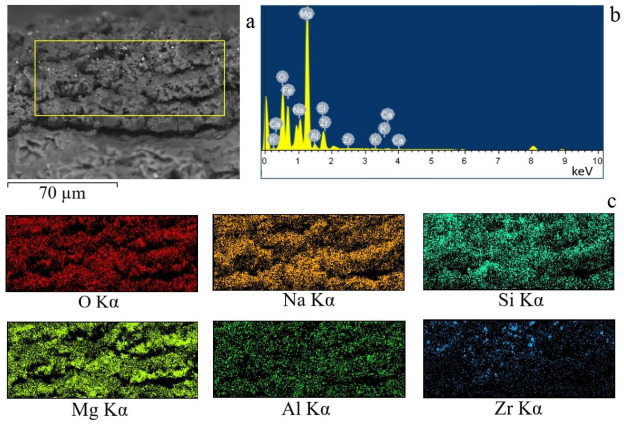
SEM image of the (**a**) cross section of the coating, (**b**) the EDX spectrum, and (**c**) the maps of element distribution in the cross-section of the coating.

The chemical analysis of the cross-section of the coating has shown that the amount of ZrO_2_ lowers towards the sample substrate (Figure 5). Only a few particles penetrate into the deeper layer of the coating, while most of them remain on the surface. All the other elements of the coating are uniformly distributed throughout the depth of the coating.

Table 2 shows the quantitative content of elements in the cross-section of the coatings deposited at different voltages of the MAO process. An increased content of oxygen, magnesium, and silicon was observed throughout the coating thickness, as well as sodium present in the coating in the amorphous phase. We would also like to note that the highest zirconium oxide content was observed in the cross-section of the coating deposited at 350 V, which correlates with the results stated above.

### 3.4. X-ray Results

**Figure 6:**
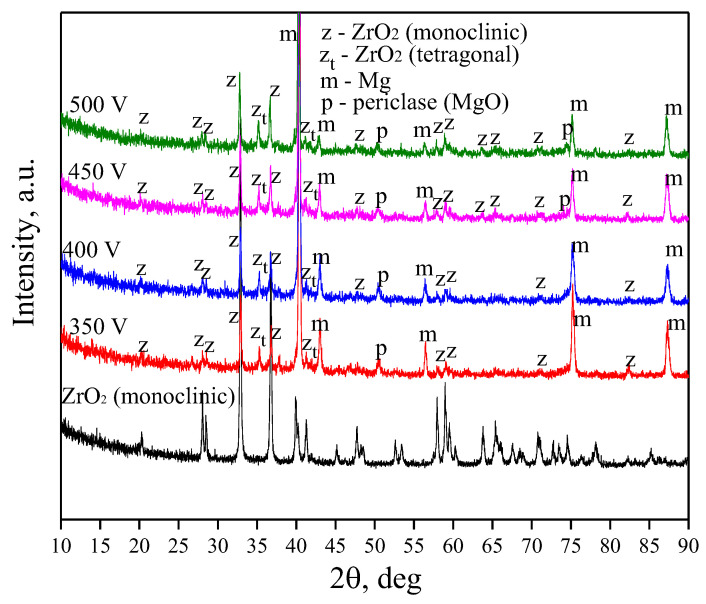
X-ray diffraction patterns of the initial ZrO_2_ powder and of the coatings obtained at different process voltages.

Figure 6 shows diffractograms of the initial zirconium dioxide powder and coatings formed at different voltages of the MAO process. X-ray diffraction of the initial powder confirms that its main crystalline phase is zirconium dioxide in the monoclinic modification. The XRD analysis of the resulting coatings has revealed that the coatings have an amorphous-crystalline structure, which is confirmed by the presence of crystalline phase reflexes and diffuse scattering in the range of 2*θ* angles from 20 to 30 degrees. The main crystalline phases identified were ZrO_2_ (monoclinic modification) (ICDD #37-1484), ZrO_2_ in tetragonal modification (ICDD #17-0923) and MgO phases (ICDD #45-0946), as well as the reflexes correlating to the pure Mg phase (ICDD #35-0821) (Figure 6) as substrate material. The coatings become more amorphous with an increase in the coating deposition voltage, which is evidenced by the lesser intensity of the peaks on the diffractograms. Interestingly, as a result of microarc oxidation, an additional high-temperature phase of zirconium dioxide in tetragonal modification is formed in the coatings. The phase transition temperature is approximately 1173 °C. The X-ray patterns show no reflexes connected with the phase formed by the interaction of diatomite with other electrolyte components or with the substrate, as described in previous studies [32]. Evidently, its melting and the formation of amorphous silicates in the structure of the coatings take place.

### 3.5. Mechanical Properties

**Figure 7:**
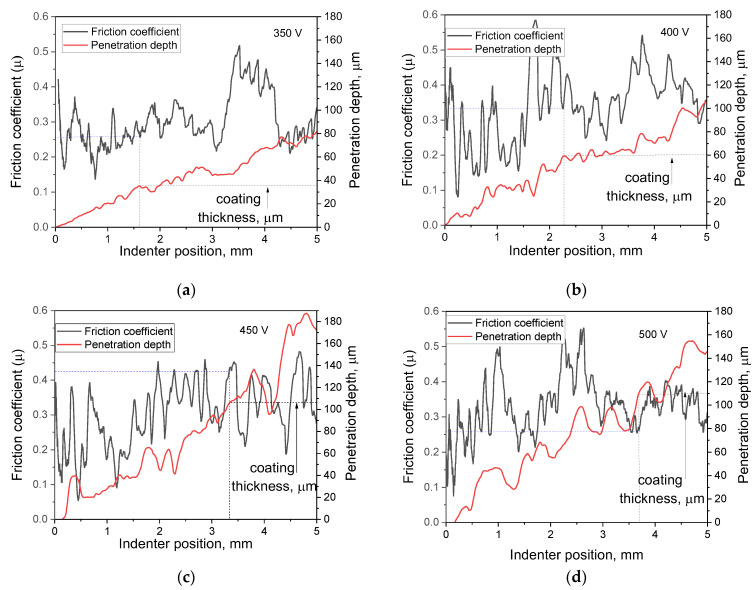
Dependence of the friction coefficient µ and the depth of penetration on the position of the indenter on the track for the coatings formed at (**a**) 350 V, (**b**) 400 V, (**c**) 450 V, and (**d**) 500 V.

Various mechanical characteristics of the resulting coatings were determined using the scratch test method. Table 3 highlights the critical load values for the coatings synthesized at different process voltages as well as the optical microscopic images of the scratches. It can be seen that the critical load value increased in accordance with the MAO voltage. The coatings deposited at 450 V have the highest critical load value and, therefore, greater adhesive and cohesive strength. However, the coating formed at a voltage of 500 V was characterized by a minimum critical load value. Figure 7 shows graphs of changes in the coefficient of friction and the depth of penetration of the indenter along the scratch for coatings formed at different voltages of the MAO process. It can be seen that the graphs have the form of oscillating curves. This is particularly characteristic of the friction coefficient graphs, which is due to the coarse topography, high roughness of the coatings, and the presence of a large number of pores and inclusions. Additional plots were made on the graphs. On the graph of the penetration depth of the indenter into the coating, the point of contact between the indenter and the substrate was determined. For this purpose, a segment equal to the coating thickness was plotted on the right Y-axis, and a perpendicular line was drawn to the graph. Then, a perpendicular was reconstructed from the obtained point to the intersection with the friction coefficient graph, and the value of the friction coefficient at this moment was determined (blue horizontal line).

It was observed that as the voltage at which the coating was applied increased from 350 to 450 V, the value of the friction coefficient at the moment of indenter-substrate contact increased from 0.26 to 0.42 (Figure 7a–c). However, for the coating formed at 500 V, this value decreased to 0.26 (Figure 7d).

It should also be noted that after the moment of hypothetical contact of the indenter with the substrate, the friction coefficient graph for the coatings applied at 350, 400, and 450 V did not change its “pulsating” character, and the amplitude of value variation practically did not change. Moreover, analyzing the optical images of the scratches on these coatings (Table 3), we can note their discontinuous character. On the contrary, for the coating formed at a voltage of 500 V, the amplitude of friction coefficient variation significantly decreases (Figure 7d), and the scratch left by the indenter on this coating is continuous. Analyzing the results of the scratch test, we can conclude that the adhesion properties of the coating obtained at a voltage of 500 V are significantly inferior to all the other coatings, despite its greater thickness. This can be explained as follows: Firstly, the number of ZrO_2_ particles in these coatings decreases (Figure 2d), which is confirmed by the results of elemental analysis (Table 1). Secondly, the number of large pores, which are concentrators of mechanical stresses, increases in these coatings. Finally, the structure of these coatings becomes more amorphous as a result of melting under the action of powerful micro-arc discharges formed at a voltage of 500 V (Figure 6).

The microhardness of the samples has also been studied. It was shown that the addition of ZrO_2_ particles generally increases the microhardness of the samples. However, it should be noted that the microhardness value decreases with the increase in MAO voltage and ranges from 137 ± 5.7 at 350 V to 80 ± 5.0 at 500 V.

### 3.6. Electrochemical Properties

**Figure 8:**
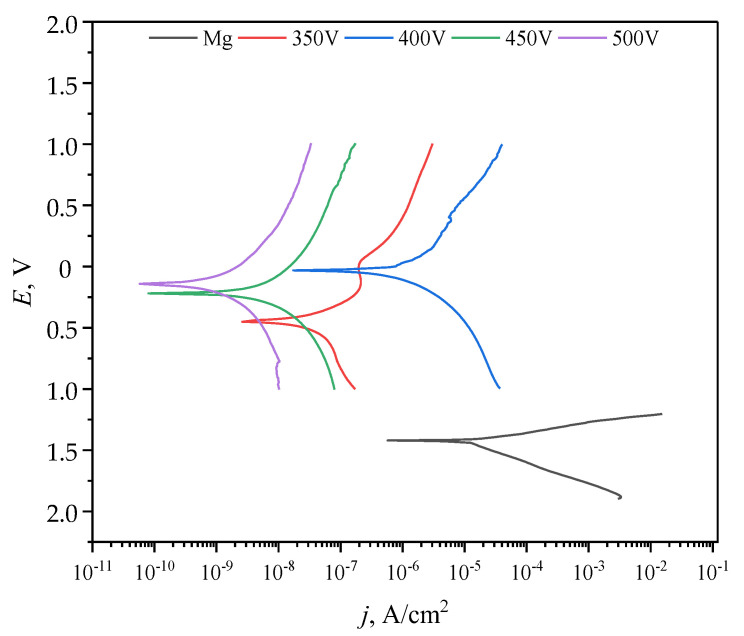
Potentiodynamic polarization (PDP) curves for the samples with and without the coating.

The electrochemical activity of both the coated and uncoated samples has been studied using the method of potentiodynamic polarization (PDP) in a 0.9% sodium chloride solution. It has been revealed that the samples coated at 450 and 500 V deposition voltages had the highest resistance to corrosion (Figure 8). The sample least susceptible to corrosion had a corrosion current density of 2.35 × 10^−10^ A cm^−2^ and a corrosion resistance of 6.34 × 10^7^ Ω cm^2^, which are 5 and 4 orders of magnitude higher than those of the uncoated Mg sample, respectively (Table 4). When comparing samples with and without the addition of ZrO_2_ particles, it was found that the addition of particles increases corrosion resistance by 2 orders of magnitude.

### 3.7. Bioresorption Process

**Figure 9:**
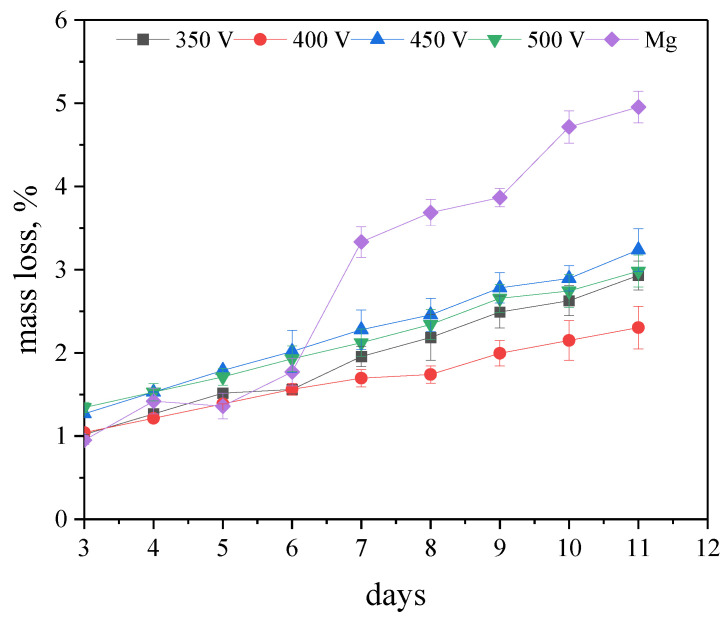
Biodegradation rate of coated samples and pure Mg in the 0.9% NaCl solution.

The bioresorption rates of both the coated and uncoated samples were compared. It was found that after 6 days of immersion in the 0.9% NaCl solution, the samples with coatings were dissolving roughly 2 times slower than Mg, and their total mass loss by the end of the experiment was in the range of 2–3% (Figure 9). The coating deposited at a 400 V MAO voltage demonstrated the lowest rate of dissolution.

**Figure 10:**
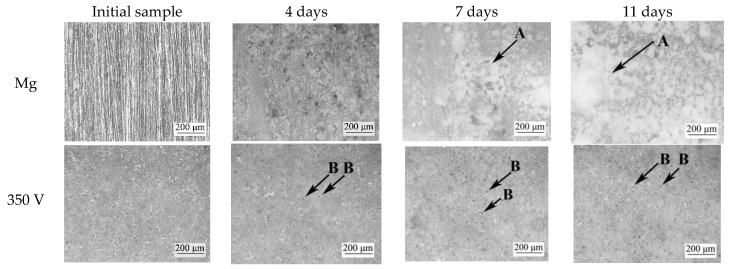

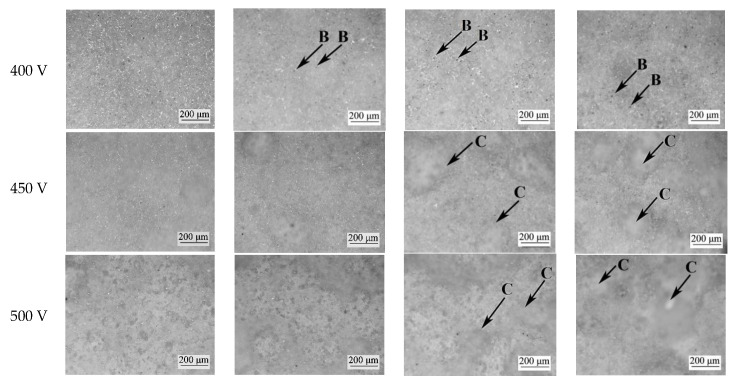
Optical microscopic images of the samples at different stages of the bioresorption process.

Visual analysis of the optical microphotographs allowed us to assess the state of the coatings and the character of their dissolution at different stages of the experiment (Figure 10). The dissolution of pure magnesium alloy results in the formation of a gel-like precipitate (Figure 10, marked with the letter A), which remains on the surface of magnesium substrates, as described in earlier studies [57] However, the process of redeposition of dissolution products does not cardinally slow down the bioresorption of magnesium alloy. Dissolution of the coatings occurs most actively in the pore area.

On the micrographs of the coatings formed at voltages of 350 and 400 V, after 4 days of dissolution, an increase in the depth and diameter of the surface pores is observed (Figure 10, marked with the letter B). For the coatings formed at higher voltages of 450 and 500 V, in addition to the above-described process, it is possible to observe redeposition of the dissolved precipitate on the surface. The micrographs show areas with gel-like deposition products. This process is most evident on the 7th and 11th days of dissolution (Figure 10, marked with the letter C).

### 3.8. In Vitro Cytotoxic Assay

**Figure 11:**
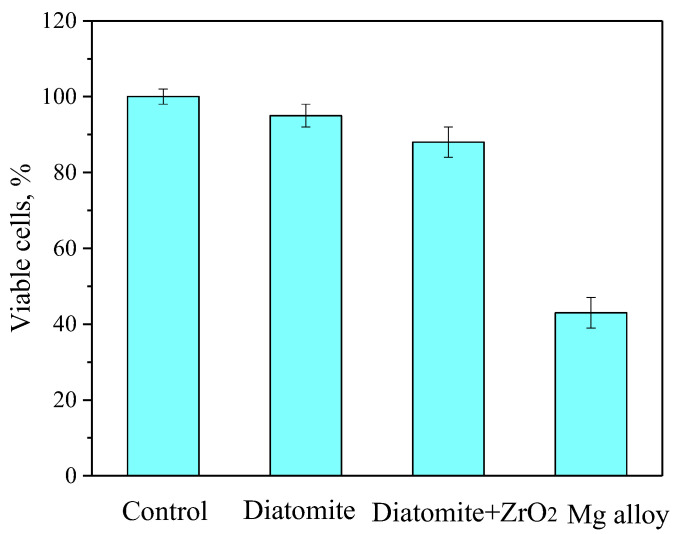
Results of in vitro NIH/3T3 cell viability measured by an MTT assay after 24-h culturing with a negative control (pure nutrient medium) as well as with Mg alloy (positive toxic control) or Diatomite and Diatomite + ZrO_2_ coatings extracts.

To reduce the number of animals involved in in vivo experiments, extensive in vitro toxicological studies were performed on appropriate cell lines in accordance with ISO 10993. Cytotoxic studies on NIH/3T3 cells were performed via the MTT test for both coated and pure magnesium alloy samples (Figure 11). When the diatomite coating extract (without ZrO_2_ particles) was added to the cell culture, a high number of viable cells was detected (95% compared to 98% in the negative cytotoxic control). Slightly fewer viable cells (88% compared to the negative cytotoxic control) were observed when interacting with the diatomite + ZrO_2_ coating extract. The Mg alloy extracts showed significant cytotoxicity according to ISO 10993-5, as the number of viable cells decreased to 43% (*p* < 0.05) after 24 hour cultivation (Figure 11).

## 4. Discussion

In recent years, a promising trend in the field of metal surface modification has been the creation of micro-arc coatings with micro- and nano-particles [58]. This provides an opportunity to create coatings with a wide range of improved functional and operational properties [59].

Many sources indicate that the behavior of particles introduced into the electrolyte and involved in the formation of coatings by micro-arc oxidation is different and depends on the melting temperature of the compound [60,61]. Particles with a high melting point remain unchanged in the coating after the micro-arc treatment. If the melting point of the particles is lower than the coating formation temperature in the MAO process, the particles melt and take part in the plasma-chemical reactions with the components of the electrolyte and substrate [62].

A novel porous biocoating based on biogenic diatomite and containing ZrO_2_ microparticles was synthesized on the surface of a high-purity Mg alloy. Such coatings can be used for modifying orthopedic implants to increase their biocompatibility and reduce their susceptibility to the influence of corrosive media. To synthesize the coatings, the method of micro-arc oxidation was used. They were found to have a complex porous structure; pores were formed both as a result of micro-arc discharges occurring on the surface during the deposition process and as a result of inheriting the reticular porous structure of diatom algae after their partial melting into the coating structure. A similar pore structure was obtained in previous studies during the formation of diatomite-based coatings [32].

It was found from SEM images that a large number of ZrO_2_ particles are evenly distributed over the surface and in the near-surface layer of coatings. However, the number of ZrO_2_ particles in the coatings decreased as the voltage of the MAO process increased. These data correlate with the results of the elemental analysis. Zirconium dioxide has a very high melting temperature of 2680 °C [56]. However, it is possible that when the voltage of the MAO process is increased to 500 V, even higher temperatures develop in the micro-arc discharge channels and the melting of zirconium dioxide particles occurs.

The analysis of the phase composition of the coatings showed the presence of an amorphous phase and crystalline phases in the coatings—zirconium oxide in monoclinic and tetragonal modifications, as well as magnesium oxide. Ceramics based on zirconium dioxide are known to have high mechanical strength, chemical resistance, and wear resistance [63,64]. In the presented study, the incorporation of ZrO_2_ particles into the diatomite coating managed to increase the mechanical strength and corrosion resistance of the coatings. While the maximum critical load for diatomite coatings without particles was 10 ± 0.8 N, for coatings with ZrO_2_ particles, this value reached 14.8 ± 0.8 N. The analysis of electrochemical parameters shows that the introduction of particles increased the corrosion resistance of the coatings by two orders of magnitude.

The study of the dissolution behavior of the coatings in the model biological liquid (0.9% NaCl solution) revealed a specific dissolution mechanism for the coatings. The dissolution of the coatings was accompanied by the process of re-precipitation of the dissolution products back onto the coating surface. As studies have shown [57], this contributes to the inhibition of the dissolution process of a coated magnesium sample.

## 5. Conclusions

The coatings were generated at four different process voltages ranging from 350 to 500 V. The thickness of the coatings ranged from 40 to 130 μm, and the roughness ranged from 3.5 μm to 11.5 μm. We have studied the effect of adding particles of refractory zirconia with a variety of research methods.

The coating formed at the process voltage of 450 V (the critical load value was 14.8 ± 0.8 N) had the highest adhesion strength. The coating formed at 500 V was characterized by high corrosion resistance. Its corrosion current density was 2.35 × 10^−10^ A cm^−2^ and its corrosion resistance was 6.34 × 10^7^ Ω cm^−2^, which were 5 and 4 orders of magnitude higher than those of the uncoated Mg sample, respectively. For the coating formed at 400 V, the lowest value of mass loss, equal to 2.3%, was recorded when it was incubated in the 0.9% NaCl solution for 11 days.

Additionally, the coatings have shown a high level of biocompatibility (i.e., low cytotoxicity) when interacting with NIH/3T3 mouse fibroblast cell culture.

## Figures and Tables

**Table 1:** Elemental composition of the coatings (at.%) depending on MAO voltage.

Element	350 V	400 V	450 V	500 V
O Kα	65.2 ± 0.9	63.9 ± 0.8	63.4 ± 1.3	63.8 ± 1.1
Na Kα	2.0 ± 0.3	2.5 ± 0.4	2.5 ± 0.5	2.8 ± 0.8
Mg Kα	15.3 ± 0.8	15.6 ± 0.7	16.7 ± 0.5	16.2 ± 0.9
Al Kα	0.7 ± 0.1	0.8 ± 0.2	0.8 ± 0.2	0.8 ± 0.4
Si Kα	12.3 ± 0.5	13.9 ± 0.9	13.5 ± 0.9	13.4 ± 0.5
Ca Kα	0.2 ± 0.1	0.1 ± 0.09	0.0	0.1 ± 0.08
Fe Kα	0.1 ± 0.03	0.2 ± 0.07	0.2 ± 0.1	0.2 ± 0.08
Zr Kα	4.2 ± 0.2	3.0 ± 0.1	2.9 ± 0.3	2.7 ± 0.2

**Table 2:** Elemental composition in the cross-section of the coatings (at.%) depending on MAO voltage.

Element	350 V	400 V	450 V	500 V
O Kα	64.0 ± 0.8	56.5 ± 1.1	56.2 ± 0.9	58.9 ± 1.1
Na Kα	1.8 ± 0.3	4.6 ± 0.6	4.1 ± 0.8	2.1 ± 0.3
Mg Kα	23.3 ± 0.3	32.5 ± 0.5	33.2 ± 0.5	32.0 ± 0.8
Al Kα	1.2 ± 0.2	1.2 ± 0.3	1.3 ± 0.5	1.5 ± 0.5
Si Kα	7.2 ± 0.3	4.5 ± 0.3	4.7 ± 0.3	4.6 ± 0.2
Ca Kα	0.2 ± 0.1	0.2 ± 0.1	0.1 ± 0.02	0.2 ± 0.1
Zr Kα	2.3 ± 0.2	0.5 ± 0.1	0.4 ± 0.1	0.7 ± 0.1

**Table 3:** Adhesive strength of coatings.

Deposition Voltage, V	Critical Load, N	Optical Microphotography
350	8.6 ± 0.5	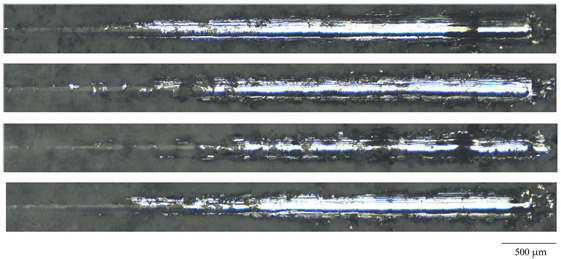
400	11.0 ± 0.6	
450	14.8 ± 0.8	
500	8.4 ± 0.7	

**Table 4:** Electrochemical parameters of the samples.

Sample	E_c_, V	J_c_, A cm^−2^	R_p_, Ω cm^2^
Mg	−1.42	1.05 × 10^−5^	1.7 × 10^3^
350 V	−0.45	1.21 × 10^−8^	1.43 × 10^6^
400 V	−0.03	7.48 × 10^−7^	0.9 × 10^4^
450 V	−0.22	1.28 × 10^−9^	1.1 × 10^7^
500 V	−0.14	2.35 × 10^−10^	6.34 × 10^7^

## Data Availability

Not applicable.
